# Emophilia: psychometric properties of the emotional promiscuity scale and its association with personality traits, unfaithfulness, and romantic relationships in a Scandinavian sample

**DOI:** 10.3389/fpsyg.2024.1265247

**Published:** 2024-04-26

**Authors:** Sol E. Røed, Randi K. Nærland, Marie Strat, Ståle Pallesen, Eilin K. Erevik

**Affiliations:** Department of Psychosocial Science, University of Bergen, Bergen, Norway

**Keywords:** infidelity, cheating, love, Big Five, Dark Triad, attraction, romantic

## Abstract

Emophilia is a suggested stable phenomenon referring to how often and easily an individual falls in love (Jones, 2011). The current study investigated the psychometric properties of the Emotional Promiscuity Scale (EPS, i.e., a measure of emophilia) and its association with personality traits, romantic relationships, and unfaithfulness in a Scandinavian sample. The sample consisted of 2,607 participants, who were recruited through Norwegian and Swedish newspapers. Descriptive analyses of the EPS and a confirmatory factor analysis (CFA) to verify the previously obtained two-factor structure were conducted. Correlations between emophilia and the Big Five and Dark Triad traits were calculated to investigate the discriminative validity of the EPS. Lastly, negative binomial regression analyses were run in which emophilia comprised the independent variable and the number of romantic relationships and number of times being unfaithful constituted the dependent variables. The EPS had satisfactory internal reliability and the responses to the items were normally distributed. The CFA indicated a two-factor structure, although the two factors correlated highly, justifying collapsing them into one dimension. Emophilia showed satisfactory discriminant validity (*r* < 00.40) against the personality traits included. Lastly, emophilia was positively associated with the number of romantic relationships and times being unfaithful. Future research should aim to improve our understanding of the psychological and behavioral aspects of emophilia.

## 1 Introduction

Many people are deeply occupied by the topic of romantic love, which is reflected in among others the endless number of movies, books, songs, and similar on the subject. Romantic love can be described as an emotion that is aimed at another individual and includes behavioral and cognitive tendencies of sexual attraction, intimacy, and caregiving (Shaver and Hazan, 1988; Fletcher et al., 2015). From an evolutionary perspective the experience of romantic love is regarded as an adaptation that motivates pair-bonding in humans, which from an evolutionary perspective is considered to be advantageous as it increases the likelihood of both parent and offspring survival (Fletcher et al., 2015). The process of going from not experiencing romantic love to experiencing it, is often conceptualized as “falling in love” (Riela et al., 2010). The phenomenon of falling in love appears to be less clearly conceptualized, as compared to romantic love (Riela et al., 2010; Cruces et al., 2015). Further, the experience of falling in love differs somewhat based on, among other things, gender, culture, attachment style, and personality (Riela et al., 2010; Cruces et al., 2015). High intensity, some level of pain, fixation, and longing appear to be common factors in many conceptualizations of falling in love (Cruces et al., 2015). Several researchers have been interested in individual differences related to falling in love (e.g., Riela et al., 2010; Cruces et al., 2015). Jones (2011) has argued that important individual differences when falling in love pertain to how easily (i.e., how rapidly) and often (i.e., how many times) one falls in love. Jones (2015) argues (and has demonstrated) that these two factors (i.e., how easily and often) reflect one intercorrelated phenomenon, which he denotes emophilia. The two factors of emophilia (i.e., easily and often) are measured by the Emotional Promiscuity Scale (EPS; Jones, 2011), but are normally collapsed into one dimension, due to the high correlation between them (Jones, 2017).

Emophilia is conceptualized as a rather stable construct, resembling a personality dimension (Jones, 2015). Jones (2011) argues that differences in how easily and how often one falls in love (i.e., emophilia) could be a consequence of differences both in how often one experiences romantic feelings and/or how often one perceives certain feelings to be romantic. Emophilia is often described as being closely linked to romantic relationship formation (Jones, 2011, 2015). The assumed tight link between emophilia and romantic relationship formation is, for instance, apparent from the comparison of emophilia and sociosexuality (Gangestad and Simpson, 1990), in which emophilia is described as the emotional/romantic equivalent of unrestricted sociosexuality (Jones, 2011, 2015). Sociosexuality is further conceptualized as being related to the number of actual sexual relationships, and not just the ease and frequency of sexual feelings (Gangestad and Simpson, 1990). Even if emophilia is closely related to romantic relationship formation, it is reasonable to expect that the association between the two constructs is not one-to-one, as people fall in love without this resulting in a romantic relationship, and conversely, form relationships without being in love.

To conclude that emophilia is indeed an important individual difference in the realm of romantic love, warrants a solid body of evidence. The research on emophilia that has been conducted is quite limited, with few studies, all of which are conducted by or in collaboration with Jones, and most of them include North American samples. Given the replication crisis in psychology (Open Science Collaboration, 2015), it is important that findings are supported by separate studies. Further, cross-cultural studies are needed to establish emophilia as a universal trait. Previous studies have found cultural differences in the perception, experience, and expression of love (de Munck et al., 2011; Jankowiak et al., 2015; Heshmati et al., 2017; Karandashev, 2021), which supports the notion of possible cultural differences in emophilia. The current study is conducted in a Scandinavian setting, and some differences between Americans and Scandinavians in the realm of love could be envisioned. De Munck et al. (2011) found for example that eastern Europeans regarded romantic feelings as more irrational and less informative, compared to Americans. As far as we know, no study has investigated whether Scandinavians also perceive feelings related to being in love as irrational compared to Americans, but if such differences exist, this might affect the associations between emophilia and romantic outcomes. It seems conceivable that individuals from cultures in which feelings related to being in love are perceived as relatively irrational might be less likely to act upon them, and hence the associations between emophilia and romantic outcomes would be expected to be weaker. Due to the limited number of studies on emophilia, especially cross-culturally, we believe it would be of interest to investigate the psychometric properties of the EPS, including its internal reliability, and to verify the factor structure in other samples (e.g., Scandinavian samples) than previously. Investigating the relationship between the EPS and personality traits and romantic outcomes could help illuminate the discriminant and predictive validity of EPS.

The associations between emophilia and personality traits could inform on the discriminant validity of the EPS. Investigation of these relationships could provide an indication of whether emophilia should be interpreted as a sub-facet of any existing personality trait (which a high correlation might suggest), or as a separate trait/phenomenon. The most acknowledged and used taxonomy of personality is currently the Big Five model, by which it is argued that personality can be understood and described by five main traits: extraversion, agreeableness, conscientiousness, neuroticism, and openness (Matthews et al., 2009). To our knowledge, two previous studies have investigated the relationship between emophilia and the Big Five traits. Jones (2017) found no significant associations between emophilia and the Big Five traits. In another study, however, Jones (2011) found an inverse association between agreeableness and emophilia, a positive association between neuroticism and emophilia among men, and a positive association between extraversion and emophilia among women. Another personality taxonomy is the Dark Triad, which refers to the personality traits of narcissism, Machiavellianism, and psychopathy (Paulhus and Williams, 2002). Narcissism is characterized by entitlement, exhibitionism, status seeking, and exploitation (Wallace et al., 2022). Machiavellianism is characterized by interpersonal manipulation and amoral viewpoints constructed to promote one’s own goals (Al Aïn et al., 2013), while psychopathy is denoted by high impulsivity and thrill seeking, and low empathy and anxiety (Paulhus and Williams, 2002). To our knowledge, only one previous study has investigated the relationship between emophilia and the Dark Triad traits, showing positive associations between emophilia and all the Dark Triad traits (Lechuga and Jones, 2021).

The predictive validity of emophilia should be established, in which romantic outcomes might be particularly relevant potential outcomes of emophilia. Emophilia has been positively associated with number of romantic relationships, marriages, divorces, marriage engagements at a younger age, pregnancies with different men, infidelity, and unrestricted and uncommitted sexual relations among women (Jones, 2011, 2015; Pinto, 2015). The relationship between emophilia and romantic outcomes may inform in terms of the predictive validity of the EPS. In addition, romantic outcomes have important individual and societal implications (Braithwaite et al., 2010; Uecker, 2012), making it important to identify potential predictors of romantic outcomes in which emophilia might play a role. Two important romantic outcomes are number of romantic relationships (as the number of marriages/engagements is declining in western societies; Schneider et al., 2018; Khan et al., 2020) and infidelity (as it involves a great deal of distress for the individuals involved; Blow and Hartnett, 2005). It is reasonable to assume that number of romantic relationships and times being unfaithful are expressions of a wide range of factors, beyond emophilia, as people fall in love without entering romantic relationships or cheating, and vice versa. Personality traits, age, and gender, among other things, have been found to be associated with romantic relationship formation and aspects of unfaithfulness (Brand et al., 2007; Altgelt et al., 2018; Erevik et al., 2020; Bozoyan and Schmiedeberg, 2023). Hence, it is important to adjust for such variables when investigating the associations between emophilia and romantic outcomes.

In summary, the current study will investigate the psychometric properties of the EPS by conducting descriptive analyses on the EPS and a confirmatory factor analysis (CFA) to verify the two-factor structure obtained in previous research (Jones, 2011). Further, to investigate discriminative validity, the study will examine the associations between emophilia and the Big Five and Dark Triad traits. Lastly, we will conduct negative binomial regression analyses to investigate the association between emophilia and number of times one has been in a romantic relationship and has been unfaithful, while adjusting for age, gender, and the Big Five and Dark Triad traits.

Based on theory and previous research the current study postulates three hypotheses. H_1_: The EPS is a psychometrically sound measure for the two-factor structure of emophilia (i.e., falling in love easily and often, respectively) and holds good psychometric properties in terms of internal reliability (alpha ≥ 0.80). H_2_: The EPS shows satisfactory discriminant validity (*r* < 0.40 with personality traits). H_3_: Emophilia shows predictive validity by being positively associated with number of romantic relationships and times being unfaithful.

## 2 Method

### 2.1 Procedure and sample

The present study utilizes data collected through the online Norwegian newspaper *VG+* and the Swedish online newspaper *Aftonbladet+.* The participants were invited to complete a digital survey via articles about emophilia published in October 2020. The sample consisted of 2,607 participants (women = 74.6%, men = 24.7%, other = 0.7%). All participants provided informed consent before completing the survey. The participants’ central tendencies on the included variables are presented in Table 1.

**Table 1:** Descriptive table.

Item	*M*	SD	%
Man			24.7
Woman			74.6
Other			0.7
Age	44.78	12.93	
Openness	15.36	3.18	
Conscientiousness	13.89	3.25	
Extraversion	13.76	3.60	
Agreeableness	17.14	2.73	
Neuroticism	12.29	3.52	
Machiavellianism	11.54	6.88	
Psychoticism	11.39	6.28	
Narcissism	18.10	7.01	
Number of romantic relationships	7.71	8.78	
Number of times being unfaithful	4.21	9.84	

M = Mean, SD = Standard Deviation.

### 2.2 Measures

#### 2.2.1 Romantic outcomes and demographics

The number of relationships and times being unfaithful were measured by asking “How many romantic relationships have you had in your life?” and “How many times have you been unfaithful?.” Response options for both variables ranged from 0 to 50 (i.e., 0, 1, 2, 3, etc.), in addition to a response option of 50+ number of relationships/times being unfaithful. Demographic variables were measured by closed-ended questions concerning age (response options ranging from 17 to 89 years (i.e., 17, 18, 19, 20, etc.) and a response option for “younger than 16” and “older than 90”) and gender (response option: “man”; “woman”; “other”).

#### 2.2.2 Dark Triad

The Dirty Dozen was used to measure the Dark Triad traits (i.e., Machiavellianism, psychopathy, and narcissism; Jonason and Webster, 2010). The questionnaire consists of 12 items (four items for each trait) with response options ranging from 1 = completely disagree to 9 = completely agree (Jonason and Webster, 2010). Thus, the total score runs on a scale from 4 to 36 for each trait. In the current study, the items measuring Machiavellianism, psychopathy, and narcissism obtained Cronbach’s alphas of 0.85, 0.74, and 0.81, respectively.

#### 2.2.3 Big Five

The Mini International Personality Item Pool (Mini-IPIP) was used to measure the Big Five personality traits (i.e., extraversion, neuroticism, openness, agreeableness, and conscientiousness; Donnellan et al., 2006). The questionnaire consists of 20 items (i.e., four items for each trait), with response options ranging from 1 = strongly agree to 5 = strongly disagree. The total score ranges from 4 to 20 for each trait. The items measuring extraversion, neuroticism, openness, agreeableness, and conscientiousness obtained Cronbach’s alphas of 0.80, 0.72, 0.71, 0.74, and 0.65, respectively, in the current study.

#### 2.2.4 Emophilia

The EPS was used to measure emophilia (Jones, 2011). The two-factored scale measures how often and easily a person falls in love and consists of 10 items. The response options range from 1 = strongly disagree to 5 = strongly agree. Although the scale has two factors, one composite score is usually derived, due to the high correlation between the two factors. The composite score ranges from 10 to 50. The items measuring emophilia obtained a Cronbach’s alpha of 0.85 in the current study.

### 2.3 Analysis

IBM Amos SPSS for Windows, version 27 (IBM Software Group, Chicago), was used to conduct a CFA to verify the two-factor structure of the EPS obtained in previous research (Jones, 2011). The error terms of items 6 and 7 were allowed to correlate based on similar wording and findings in previous research (Jones, 2011). A recommended cut-off value of >0.32 for factor loading values was used (Worthington and Whittaker, 2006). The model fit was assessed by the comparative fit index (CFI), the Tucker-Lewis index (TLI), and the root mean square error of approximation (RMSEA; including 90% confidence interval), in which cut-off values of 0.90, 0.90, and 0.10 signify acceptable fit, respectively (Byrne, 2012). Further, the two-factor structure was compared to a hierarchical-, bifactor-, and single factor model to assess the best fitting structure for the EPS. The models were compared based on the Akaike Information Criterion (AIC) in which a lower AIC indicates a better model parsimony (Bonakdari and Zeynoddin, 2022). The model fit was also assessed by CFI, TLI, and RMSEA. The measurement invariance of gender (i.e., men and women) and age groups (i.e., 35 or younger, 36–55, and 56 or older) were measured through multigroup confirmatory factor analysis (MGCFA). The 35 or younger group consisted of 704 participants (women = 74.6%, men = 24.6%, other = 0.8%), and the 36 to 55 group consisted of 1,356 participants (women = 76.8%, men = 22.5%, other = 0.7%), while the 56 or older group consisted of 546 participants (women = 69.0%, men = 30.6%, other = 0.4%). The configural invariance was assessed by CFI, TLI, and RMSEA and the metric invariance was assessed by comparing the metric and configural model, where a non-significant result would indicate equivalence (Byrne, 2012).

The other analyses were conducted using IBM SPSS for Windows, Version 27 (IBM Corp., Armonk: NY). Descriptive analyses were conducted to obtain an overview of the data and investigate the distribution and reliability of the EPS. Further, correlation analyses were conducted to investigate correlations between emophilia and the traits in the Big Five and Dark Triad models, age, and gender. *p*-values < 0.05 were used to indicate statistical significance. The correlations were reported in terms of Pearson’s *r* (except for the relationship with gender, which was expressed in terms of a point-biserial correlation coefficient). Correlation coefficients of 0.1, 0.3, and 0.5 are considered to be small, medium, and large effect sizes, respectively (Cohen, 1988).

The data did not meet the criteria of a poisson regression analysis due to overdispersion (Lærd Statistics, 2018). Therefore, negative binomial regression analyses were conducted to investigate the association between the independent variable “emophilia” and the dependent variables “number of relationships” and “number of times unfaithful.” The variable age was included as an offset variable. Age was entered as a continuous adjustment variable.

Crude, partly, and fully adjusted regression analyses were conducted. The Big Five traits (i.e., extraversion, neuroticism, openness, agreeableness, and conscientiousness), Dark Triad traits (i.e., Machiavellianism, psychopathy, and narcissism), age, and gender were controlled for in a stepwise manner. The associations between emophilia and the dependent variables were reported in terms of incident rate ratio (IRR). IRRs of 1.22, 1.86, and 3.00 indicate small, medium, and large effect sizes, respectively (Cohen, 1988; Olivier et al., 2017).

## 3 Results

Results from descriptive analyses on the EPS are displayed in Table 2. Items 1–9 had a skewness and kurtosis between −2 and +2. This is considered acceptable for a univariate normal distribution (Curran et al., 1996). Variable 10, “how many people have you fallen in love with?,” had a skewness-score below −2 and a kurtosis-score above +2.

**Table 2:** Descriptive statistics of the EPS.

Item	*M*	SD	Skewness	Kurtosis	α
**Easily**					0.80
1. I fall in love easily	3.20	1.16	−0.21	−0.79	
2. For me, romantic feelings take a long time to develop	3.41	1.08	−0.46	−0.51	
3. I feel romantic connections right away	3.26	1.13	−0.32	−0.82	
4. I love the feeling of falling in love	3.68	1.04	−0.69	0.04	
5. I am not the type of person who falls in love	3.94	1.03	−0.86	0.15	
**Often**					0.74
6. I often feel romantic connections to more than one person at a time	2.29	1.17	0.57	−0.72	
7. I have been in love with more than one person at the same time	2.67	1.36	0.16	−1.40	
8. I fall in love frequently	2.65	1.12	0.27	−0.80	
9. I tend to jump into relationships	2.77	1.30	0.14	−1.20	
10. How many people have you fallen in love with?	4.66	0.74	−2.42	5.68	
Composite emophilia score	32.55	7.39	−0.05	−0.42	0.85

M = Mean, SD = Standard Deviation, a = Cronbach’s alpha.

**Figure 1:**
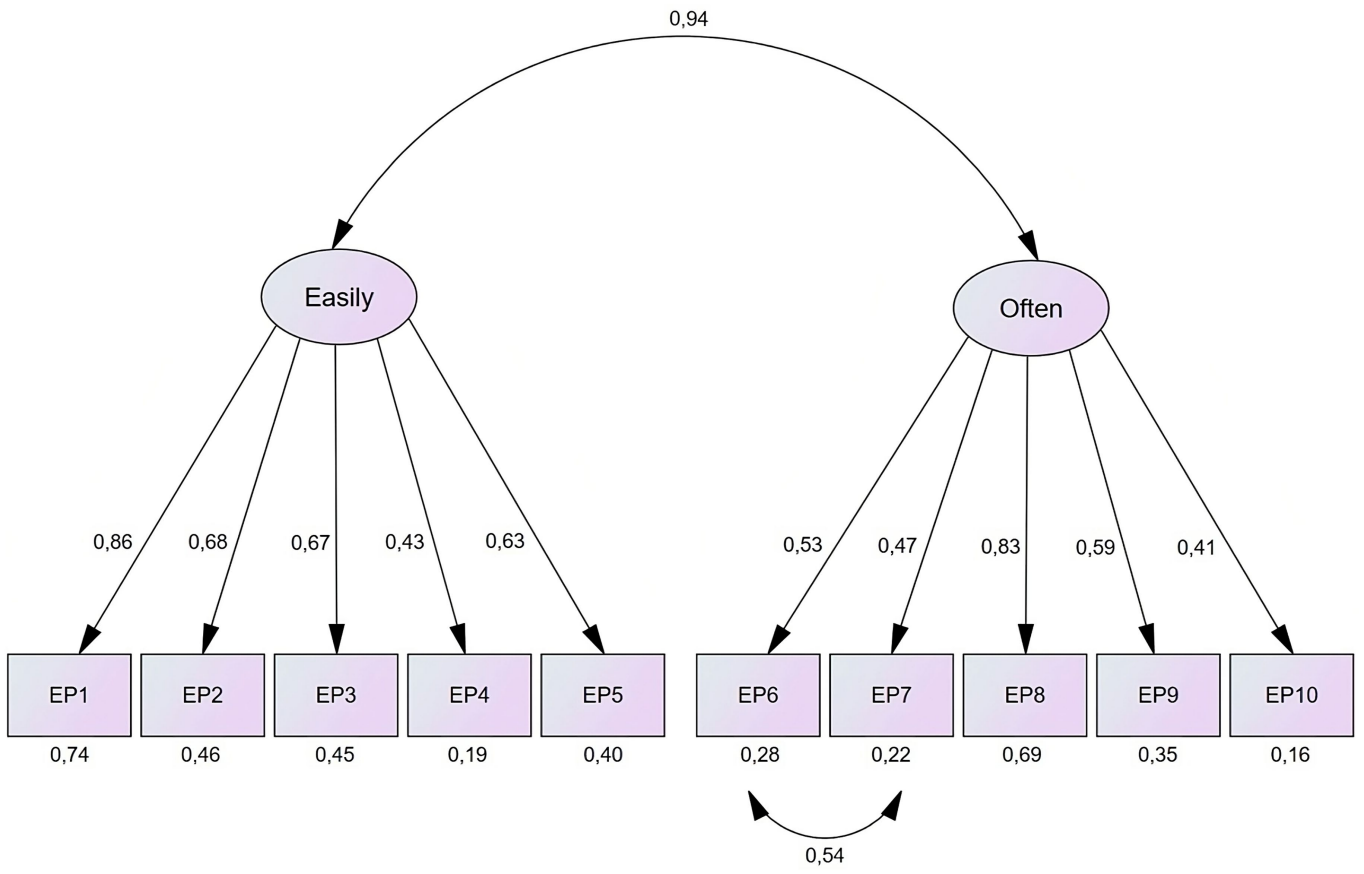
The Two-Factor Structure of the EPS.

Results from the CFA conducted on the EPS are presented in Figure 1. The model fit was assessed by three indexes (i.e., CFI = 0.95, TLI = 0.91, and RMSEA = 0.072, 90% *CI* RMSEA [0.067–0.078]), which indicated a moderate to good fit (Byrne, 2012). Items 1–5 loaded on factor 1 (easily) and items 6–10 loaded on factor 2 (often). The error term of items 6 and 7 had a correlation of 0.54. We ran a sensitivity analysis to make sure that allowing the two error terms to correlate did not inflate the factor loadings. Running a model without the correlation between the two error variances marginally changed the factor loading of EP6 (from 0.53 to 0.59) and EP7 (from 0.47 to 0.54).

We also tested competing models with a hierarchical-, bifactor-, and single factor structure. The models were assessed by comparing AIC. The AICs for the two-factor model, hierarchical model, bifactor model and single factor model was 556, 592, 385 and 1,435, respectively. This indicates that the bifactor model has the best fitting structure for the EPS (Bonakdari and Zeynoddin, 2022).

**Figure 2:**
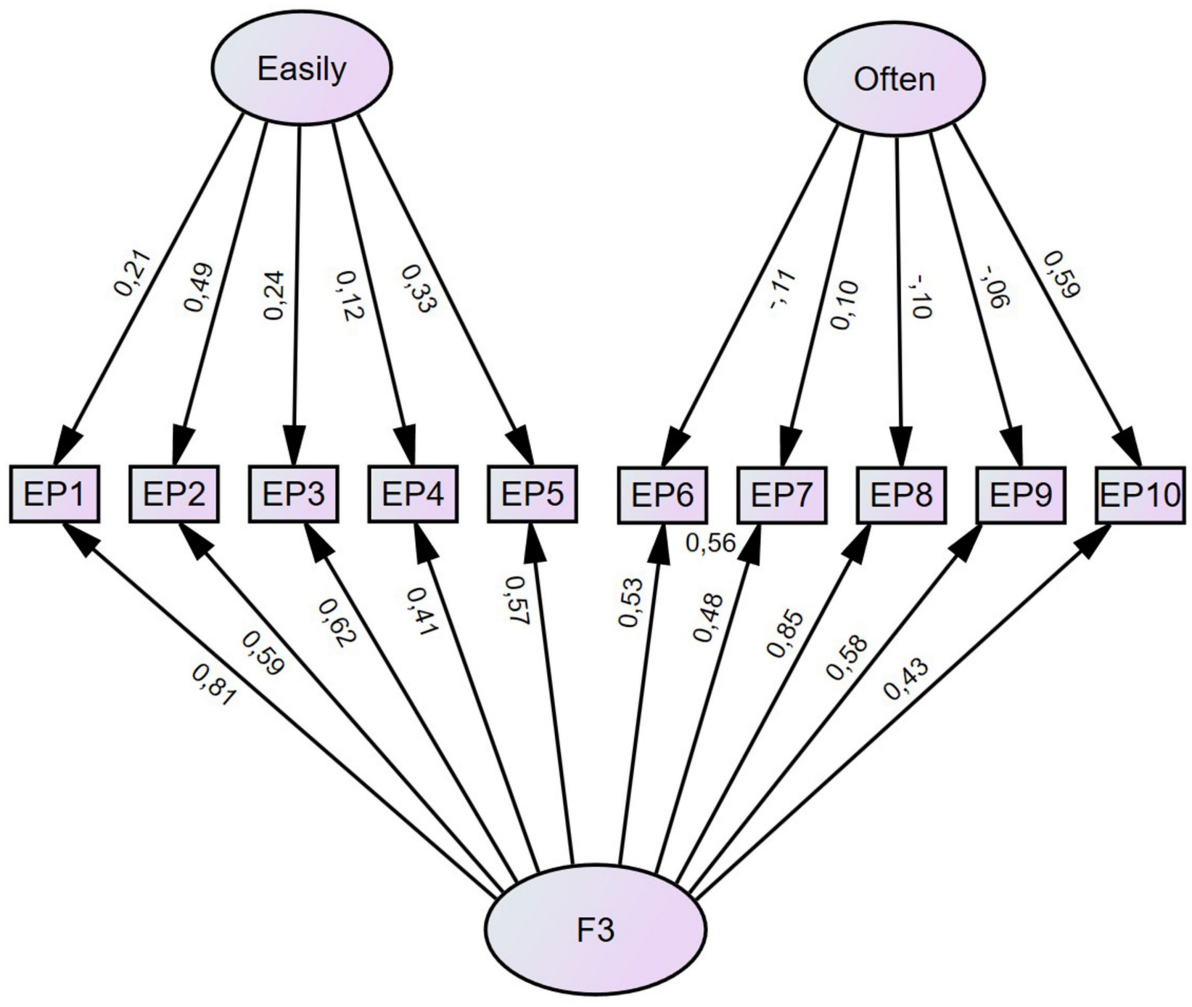
The Bifactor Structure of the EPS.

The model fit for the competing models were also assessed through CFI, TLI, and RMSEA. The hierarchical model had a moderate to good fit (CFI = 0.94, TLI = 0.91, RMSEA = 0.074 [0.069–0.080]). The results for the single factor model indicated poor fit (CFI = 0.85, TLI = 0.76, RMSEA = 0.120 [0.114–0.125]). Lastly, the bifactor model had a moderate to good fit (CFI = 0.97, TLI = 0.93, RMSEA = 0.066 [0.060–0.073]) (Byrne, 2012). Overall, the bifactor model was the best fitting model for the EPS and are presented in Figure 2.

The MGCFA conducted on the bifactor model across gender (CFI = 0.97, TLI = 0.94, RMSEA = 0.042) and age groups (CFI = 0.97, TLI = 0.93, RMSEA = 0.038) indicated a moderate to good model fit (Byrne, 2012). However, the metric analysis of the age groups obtained a significant result (*p* < 0.000) and therefore did not meet the criterion of metric invariance (Byrne, 2012).

The zero-order correlations between emophilia and the Big Five and Dark Triad traits, age, and gender are presented in Table 3. Emophilia had a small inverse correlation with conscientiousness and a small positive correlation with extraversion, agreeableness, neuroticism, and openness. Furthermore, emophilia had small positive correlations with Machiavellianism and psychopathy, and a medium positive correlation with narcissism. The association between emophilia and gender was not statistically significant, and emophilia had a small inverse correlation with age.

**Table 3:** Pearsons’ Correlations.

	1	2	3	4	5	6	7	8	9	10	11
1. Emophilia											
2. Age	−0.08^**^										
3. Extraversion	0.14^**^	−0.04									
4. Agreeableness	0.06^**^	0.05^*^	0.22^**^								
5. Conscientiousness	−0.17^**^	0.11^**^	0.01	0.07^**^							
6. Neuroticism	0.25^**^	−0.19^**^	−0.06^**^	0.03	−0.17^**^						
7. Openness	0.09^**^	0.10^**^	0.16^**^	0.17^**^	−0.10^**^	0.01					
8. Narcissism	0.34^**^	−0.21^**^	0.23^**^	−0.13^**^	−0.07^**^	0.16^**^	0.02				
9. Psychopathy	0.14^**^	−0.01	0.03	−0.45^**^	−0.14^**^	0.01	−0.01	0.33^**^			
10. Machiavellianism	0.28^**^	−0.12^**^	0.11^**^	−0.28^**^	−0.18^**^	0.15^**^	0.02	0.48^**^	0.53^**^		
11. Gender^a^	0.01	0.05^*^	−0.06^**^	−0.22^**^	−0.06^*^	−0.16^**^	0.02	0.05^*^	0.19^**^	0.11^**^	

Pearson’s correlations. ^*^p < 0.05, ^**^p < 0.01.

a Point-biserial correlation coefficient.

Negative binomial regression analyses were conducted in which emophilia was the independent variable and number of romantic relationships comprised the dependent variable. The results are presented in Table 4. Age, gender, and the Big Five and Dark Triad traits were adjusted for in a stepwise manner. Emophilia had a positive association with the variable number of romantic relationships in all regression analyses (*p* < 0.001). The effect sizes of the associations between emophilia and number of romantic relationships were small in all analyses. Adjusting for all the variables included did not appear to affect the association between emophilia and the dependent variable, as the confidence interval of the crude effect overlapped with the confidence intervals of the adjusted effects.

**Table 4:** Negative binomial regression on the relationship between emophilia and number of romantic relationships.

Independent variables	IRR (95% CI)
EmophiliaZ (crude) *Model (crude)*	1.22 (1.18–1.26)^***^ Chi square = 145.68 *df* = 1
EmophiliaZ (adjusted for gender and age)	1.20 (1.17–1.24)^***^
EmophiliaZ (adjusted for Big Five)	1.21 (1.17–1.26)^***^
EmophiliaZ (adjusted for Dark Triad)	1.19 (1.15–1.24)^***^
EmophiliaZ (fully adjusted)	1.21 (1.17–1.25)^***^

IRR = Incident Rate Ratio, CI = Confidence Interval, Z = Z-Score, df = Degrees of freedom, ^***^p < 0.001.

Further, negative binomial regression analyses in which emophilia was the independent variable and unfaithfulness constituted the dependent variable were conducted. The results are presented in Table 5. The variables age, gender, and Big Five and Dark Triad traits were adjusted for in a stepwise manner. Emophilia had a positive association with unfaithfulness at all steps (*p* < 0.001). The effect sizes of the associations between emophilia and unfaithfulness were small in all analyses. The confidence interval of the crude effect overlapped with the confidence intervals in which age, gender, Big Five, and Dark Triad were adjusted for. However, the confidence interval of the crude effect did not overlap with the confidence interval of the effect in the fully adjusted analysis, suggesting that the effect was somewhat weakened when all the variables were adjusted for.

**Table 5:** Negative binomial regression on the relationship between emophilia and number of times being unfaithful.

Independent variables	IRR (95% CI)
EmophiliaZ (crude) *Model (crude)*	1.43 (1.36–1.50)^***^ Chi-square = 189.04 df = 1
EmophiliaZ (adjusted for gender and age)	1.40 (1.33–1.47)^***^
EmophiliaZ (adjusted for Big Five)	1.37 (1.30–1.45)^***^
EmophiliaZ (adjusted for Dark Triad)	1.30 (1.24–1.38)^***^
EmophiliaZ (fully adjusted)	1.25 (1.18–1.32)^***^

IRR = Incident Rate Ratio, CI = Confidence Interval, Z = Z-score, df = Degrees of freedom, ^***^p < 0.001.

The variance inflation factor was 1.26 in the analyses concerning both number of romantic relationships and unfaithfulness; hence multicollinearity was not present. The current study also investigated whether gender was a moderator in the relationships between emophilia and number of romantic relationships and times being unfaithful. No statistically significant interaction effects were found (results not shown).

## 4 Discussion

The current study investigated the psychometric properties of the EPS. The descriptive analyses and factor analysis suggested that the EPS had a satisfactory internal reliability, that responses to the items were normally distributed, and that the EPS had a similar factor structure to the one obtained in previous research (Jones, 2011). The effect sizes of the associations between emophilia and the other variables (i.e., gender, age, personality traits, and romantic outcomes) were small in most cases, suggesting both acceptable discriminative validity and some, but limited, predictive validity. It should, however, be noted that effect sizes are often small within the field of individual differences (Gignac and Szodorai, 2016), and hence some of the observed associations might be closer to medium in size, compared to common benchmarks. In the following, the current findings will be discussed in relation to our hypotheses and findings from previous studies. Further, we will discuss whether emophilia could be a potential personality trait.

H_1_ [the EPS is a psychometrically sound measure for the two-factor structure of emophilia (i.e., falling in love easily and often, respectively) and holds good psychometric properties in terms of internal reliability (alpha ≥ 0.80)] was supported. The results indicated a two-factor structure (although they could be collapsed into one dimension due to a high correlation between the factors) and a satisfactory internal reliability of the EPS.

H_2_ [the EPS shows satisfactory discriminant validity (*r* < 0.40 with personality traits)] was supported. The correlation analyses indicated discriminant validity against personality traits. The effect sizes of the associations between emophilia and the personality traits included were mostly small, except for the association with narcissism, which had a medium effect size. The small effect sizes suggest that emophilia is not completely explained by existing personality traits. However, some personality facets do not have strong correlations with their corresponding traits (Le Corff and Busque-Carrier, 2016), and thus it might still be reasonable to incorporate emophilia in an existing personality trait if there are sound theoretical rationales for this. Further, emophilia might still be the result of a combination of traits and/or other factors not measured in the current study.

H_3_ (Emophilia shows predictive validity by being positively associated with the number of romantic relationships and times being unfaithful) was supported. The association between emophilia and romantic relationships may be explained by the tendency to fall in love easily and often (Jones, 2011). Further, this might lead the individual to engage in new romantic relationships more frequently. Falling in love easily and often may also explain emophilia’s association with unfaithfulness, as it may lead the individual to develop romantic feelings toward someone outside their relationship, which may contribute to them being unfaithful. In the current study, the effect sizes of the associations between emophilia and number of relationships and unfaithfulness were small, which supports the notion that romantic outcomes are determined by a range of factors, in addition to one’s tendency to fall in love easily and often. The current study is cross-sectional and based on self-reporting, and hence it might be that instead of emophilia causing the number of relationships/affairs, the direction could be opposite, in which scores on emophilia were at least in part a consequence of the number of relationships/affairs. One can reason that those who have been in many relationships, and/or cheated many times, might reason in hindsight that they might also have been in love many times, as it is common, and it is probably more socially desirable, to view relationship formation/cheating as being related to love. Further, the associations found between emophilia and number of relationships/unfaithfulness might also be explained by common third variables. In the current study we sought to adjust for the effect of some potential third variables (i.e., gender, age, and personality traits) by adjusting for them in the analyses. It is, however, important to note that interpreting findings from adjusted analyses is not straightforward, as adjustment variables should only be included if they are real third variables; whereas including adjustment variables that are in part or completely caused by the independent and/or dependent variable could mask or distort the actual relationship (Spector and Brannick, 2011; Elwert and Winship, 2014). In the current study, it is possible that the personality variables, in particular, might in part be consequences of emophilia and/or the relationship outcomes. Therefore, it is recommended to interpret the results of the adjusted analyses with caution.

Overall, the current findings appear to be in line with previous findings, which suggest that emophilia might be similar in Scandinavia as in North America. The results supported a two-factor structure in the EPS similar to what has been obtained in previous research (Jones, 2011). The obtained Cronbach’s alpha of the EPS was also similar to those obtained in previous studies (Jones, 2011), which suggests that the EPS has similar internal reliability in North American and Scandinavian samples. Further, emophilia generally had similar associations with the Big Five and Dark Triad traits in the current study to those found in previous studies, both in terms of size and direction (Jones, 2011, 2017; Lechuga and Jones, 2021). An exception was agreeableness, however, which was positively associated with emophilia in the current study, but inversely associated with emophilia in previous research (Jones, 2011, 2017). Hence, there might be a cultural difference between the American and Scandinavian population in terms of the relationship between agreeableness and emophilia. Possible explanations for this discrepancy are not apparent. Our findings concerning emophilia and romantic outcomes, in this case romantic relationship formation and cheating, are also in line with previous findings in terms of direction (Jones, 2011, 2015; Pinto, 2015). Hence, emophilia may have similar descendants in North America and Scandinavia. In the current study, the associations between emophilia and number of relationships and infidelities had small effect sizes, while previous studies have reported medium effect sizes for these associations. The strength of the relationship between emophilia and romantic outcomes might thus be weaker in Scandinavia, but we used another effect size indicator compared to previous studies, and the effect sizes might thus not be directly comparable. It is important to note that even though the current findings in general support that emophilia is a similar phenomenon in Scandinavia and North America, the current methods preclude conclusions regarding cultural comparability, as no statistical comparisons between samples from different countries were made. Further, there are several other parameters that should be investigated cross-culturally before drawing conclusions on the cultural equivalence of emophilia, e.g., potential differences in thresholds for what is considered being in love.

Results from the present study indicate that the EPS has good psychometric properties. However, there is a need for more research to draw conclusions as to whether emophilia should be regarded as a stable, unique trait or as a temporary state/outcome depending on a range of other factors. For one, longitudinal studies should be conducted to determine the temporal stability of emophilia. Still, even if stability is established, emophilia might instead be a consequence of a range of other factors, some of which might be rather stable. Important factors which have been found to predict emophilia or related constructs include attachment style (Tosun et al., 2021; Frowijn et al., 2022), cognitive factors (e.g., self-representation; Quintard et al., 2020; Bouquet et al., 2021), and loneliness (Goh et al., 2023). Further, it is reasonable to expect that both life stage, in terms of age and relationship status/relationship satisfaction, and life experiences, in particular in the romantic domain, may affect how easily and often one falls in love. For instance, Collibee and Furman (2016) demonstrated how sexual experience was associated with romantic cognitions, which may further determine ease and frequency of falling in love. One could also speculate that emophilia might be a consequence of an unstable life situation and/or personality structure, in which the individual is searching for a partner (among other things). Entering a romantic relationship has further been suggested to be associated with both increased environmental and personality stability, and personality maturation (Lehmann et al., 2013; Briley and Tucker-Drob, 2014; Wagner et al., 2015; Bleidorn et al., 2018; Bühler et al., 2023). Genotype may also affect emophilia, as romantic outcomes are influenced by genes (Whisman and South, 2017). In summary, emophilia might reflect a behavioral outcome resulting from a complex interplay of genetic, environmental, cognitive, and personality factors. Differentiating potential personality traits (e.g., emophilia) from behavioral outcomes that could be an expression of personality traits (i.e., characteristic adaptations) is no simple task, especially considering that characteristic adaptations have not yet been clearly operationalized (Henry and Mõttus, 2020).

### 4.1 Limitations

The current study investigated the association between emophilia and romantic relationships and unfaithfulness. However, there was no definition of the terms unfaithful and romantic relationship in the questionnaire and these concepts may therefore have been perceived differently by the different participants. Hence, it is possible that the observed associations are reflections of the individuals who scored high on emophilia simply defining love and unfaithfulness in a way that made them achieve higher numbers.

The study sample was based on convenience sampling, which could lead to selection bias and, as such, limit the generalizability of the current findings. For instance, it is reasonable to assume that people who had heard of or were interested in emophilia and/or related concepts would have been more likely to respond to the survey. Individuals with an increased interest in emophilia might differ from others in several ways, and a potential overrepresentation of such individuals might thus have affected the results. Further, the newspaper articles were only available online and for paying subscribers. This may have excluded the elderly population and people with fewer financial means, which could also have affected the generalizability of the results.

As the study was based on self-report only and used a cross-sectional design, factors such as recall bias (Talari and Goyal, 2020), social desirability bias (Krumpal, 2013), and the common method bias (Podsakoff et al., 2003) may have influenced the findings. The fact that metric invariance for the EPS across age groups was not found is also a limitation.

A final important limitation we would like to mention is that several important constructs which might have elucidated emophilia better, e.g., attachment style, cognitive factors, and life events, were unfortunately not included in the current study.

### 4.2 Implications for further studies

Further studies addressing the findings of the present study should consider the limitations mentioned. Particularly important is the need to operationalize the terms romantic relationship and unfaithfulness, as they can be interpreted idiosyncratically. Additionally, several interesting questions remain for future research. Future research should aim to improve our understanding of the psychological and behavioral aspects of emophilia. For instance, an exploration of the relationship between emophilia and sense of agency (e.g., in terms of perceived control over romantic actions), and other cognitive processes (e.g., self-concept clarity) could offer new insight. Another interesting inquiry for future research could be to investigate the relationship between emophilia and susceptibility to persuasion and external influences (Di Plinio et al., 2023). Lastly, to establish emophilia as a universal stable trait it is necessary to conduct both cross-cultural and longitudinal studies, and to elucidate how emophilia is related to several important constructs and theories within the field of romantic love (e.g., Tobore, 2020). Cross-cultural studies should make direct comparisons between samples, include variables that might be particularly relevant for cross-cultural comparisons, and have a solid theoretical foundation.

## 5 Conclusion

The present study indicates that the EPS holds good psychometric properties. Emophilia showed satisfactory discriminant validity (*r* < 0.40) against the personality traits included. Lastly, the study indicates that emophilia may be associated with entering more romantic relationships and unfaithfulness, but the cross-sectional design of the current study precludes conclusions concerning directionality. Future research should aim to improve our understanding of the psychological and behavioral aspects of emophilia. More research, including both longitudinal and cross-cultural studies, is also needed to establish emophilia as a personality trait.

## Data availability statement

The raw data supporting the conclusions of this article will be made available by the authors, without undue reservation.

## Ethics statement

The requirement of ethical approval was waived by Forsknings- og forskerutdanningsutvalget/Research and Research Training (FFU) University of Bergen for the studies involving humans because the project utilizes data collected anonymously and does not handle personal information. The studies were conducted in accordance with the local legislation and institutional requirements. The participants provided their written informed consent to participate in this study.

## Author contributions

SR: Writing – original draft, Writing – review & editing. RN: Writing – original draft. MS: Writing – original draft. SP: Supervision, Writing – review & editing. EE: Project administration, Supervision, Writing – review & editing.
